# Pancreaticoduodenectomy for Mass-Forming Localized Duodenal Amyloidosis: A Case Report

**DOI:** 10.70352/scrj.cr.26-0447

**Published:** 2026-10-06

**Authors:** Kenichiro Yambe, Kei Nakagawa, Yoko Okitsu, Masashi Kato, Fumiyoshi Fujishima, Kuniharu Yamamoto, Hiroto Sakurai, Kazuhiro Takami, Noriko Kondo, Nobuhiro Hasui, Riki Ujihara, Yu Katayose

**Affiliations:** 1 Division of Hepato-Biliary and Pancreatic Surgery, Tohoku Medical and Pharmaceutical University, Sendai, Miyagi, Japan; 2 Division of Hematology and Rheumatology, Tohoku Medical and Pharmaceutical University, Sendai, Miyagi, Japan; 3 Division of Diagnostic Pathology, Tohoku Medical and Pharmaceutical University, Sendai, Miyagi, Japan

**Keywords:** duodenal amyloidosis, ALλ amyloidosis, localized amyloidosis, mass-forming amyloidosis, gastrointestinal amyloidosis, pancreaticoduodenectomy

## Abstract

**INTRODUCTION:**

Localized duodenal amyloidosis is extremely rare, with only a few cases having been documented in the literature. To the best of our knowledge, we report the first case of localized amyloid light-chain (AL) λ-type duodenal amyloidosis diagnosed after pancreaticoduodenectomy (PD).

**CASE PRESENTATION:**

A 71-year-old asymptomatic man was referred to our hospital after plain radiography revealed a calcified mass in the upper abdomen. Contrast-enhanced CT revealed a 58-mm exophytic mass with prominent peripheral calcification on the dorsal aspect of the descending duodenum, showing continuity with the pancreatic head vasculature. Endoscopic ultrasonography (EUS) could not evaluate the internal structure because of acoustic shadowing from dense calcification, thereby precluding EUS-guided tissue acquisition. Because malignancies, including gastrointestinal stromal tumors and lymphoma, could not be excluded, a subtotal stomach-preserving PD was performed for diagnostic and therapeutic purposes. Histopathological examination revealed extensive amyloid deposition that tested positive for Congo red staining, direct fast scarlet, and λ-light chain, while showing negative staining for κ-light chain, amyloid transthyretin, and serum amyloid A, thereby establishing a diagnosis of localized ALλ-type amyloidosis. A comprehensive systemic workup, including echocardiography, cardiac MRI, serum and urine immunofixation electrophoresis, and free light chain assays, excluded systemic amyloidosis. The patient was managed with observation alone without chemotherapy and remained recurrence-free for 2 years postoperatively.

**CONCLUSIONS:**

When preoperative tissue diagnosis is not feasible because of extensive calcification and malignancy cannot be excluded, surgical resection, including PD, for localized ALλ-type duodenal amyloidosis should be considered as both a diagnostic and therapeutic intervention. After a diagnosis of localized amyloidosis is made, the exclusion of systemic disease and long-term follow-up are essential to monitor for local recurrence, the emergence of clonal disease, and potential progression to systemic disease.

## Abbreviations

AA
: amyloid A
AL
: amyloid light-chain
ALT
: alanine aminotransferase
AST
: aspartate aminotransferase
ATTR
: amyloid transthyretin
CA19-9
: carbohydrate antigen 19-9
CEA
: carcinoembryonic antigen
DFS
: direct fast scarlet
EGD
: esophagogastroduodenoscopy
EUS
: endoscopic ultrasonography
EUS-TA
: endoscopic ultrasound-guided tissue acquisition
GIST
: gastrointestinal stromal tumor
PD
: pancreaticoduodenectomy
SAA
: serum amyloid A
SMM
: smoldering multiple myeloma

## INTRODUCTION

Amyloidosis comprises a group of diseases in which misfolded precursor proteins deposit extracellularly as amyloid fibrils, disrupting tissue architecture and organ function.^1)^

It is classified according to the causative precursor protein, and the AL, AA, and ATTR types account for most cases.^2)^ AL amyloidosis occurs in both systemic and localized forms; systemic disease may involve the heart, kidneys, liver, and nerves, whereas localized disease is confined to a single organ.^3)^

Localized amyloidosis of the duodenum is extremely rare, and mass-forming lesions are exceptional; to the best of our knowledge, no case of localized duodenal amyloidosis managed by PD has been reported. Herein, we present a case of localized ALλ-type duodenal amyloidosis that presented as a densely calcified duodenal mass. The take-home message of this report is a practical one regarding diagnostic and surgical decision-making: when dense calcification precludes EUS-TA and a malignant tumor such as a GIST cannot be excluded, surgical resection may be justified as a combined diagnostic and therapeutic procedure; once localized amyloidosis is diagnosed, systemic disease must be excluded and long-term surveillance must be maintained.

## CASE PRESENTATION

### Patient background and clinical course

A 71-year-old man was referred to our hospital for further evaluation after a calcified upper abdominal mass was identified on plain radiography. His medical history included an appendectomy and benign prostatic hyperplasia. He was asymptomatic.

### Preoperative investigations

EGD demonstrated a submucosal tumor-like elevation with smooth overlying mucosa (**Fig.1A**). EUS could not evaluate the internal structure because of acoustic shadowing from the dense calcification, and a safe puncture route could not be secured; therefore, EUS-TA was not feasible (**Fig. 1B**). Contrast-enhanced CT revealed a 58-mm exophytic mass on the dorsal aspect of the descending duodenum with prominent peripheral calcification and contrast enhancement. The mass was located in the retroperitoneum and predominantly exhibited dense peripheral calcification. The mass shared the vascular distribution of the duodenum, and continuity with the pancreatic head arcade vessels was noted, suggesting a duodenal origin (**Fig. 1C**). Contrast-enhanced MRI revealed low signal intensity on T2-weighted images at the periphery, consistent with calcification, and no fatty signal components were identified. Diffusion-weighted imaging showed faint hyperintensity at the periphery and subtle hypointensity on the apparent diffusion coefficient map (**Fig. 1D**, **1E**). A dynamic contrast-enhanced study revealed progressive centripetal enhancement of the interior of the mass, with the contrast effect gradually expanding from the periphery toward the center (**Fig. 1F**–**1H**). Based on these imaging findings—a retroperitoneal, exophytic mass showing vascular continuity with the duodenum, dense peripheral calcification, absence of a fatty signal, and progressive centripetal enhancement—a tumor of duodenal origin was suspected, and a mesenchymal tumor, in particular GIST, was considered the most likely diagnosis. However, neither the calcification pattern nor the enhancement dynamics was sufficiently specific to exclude duodenal carcinoma, neuroendocrine tumor, or lymphoma. Preoperative histological diagnosis was attempted but could not be obtained: the intact overlying mucosa meant that forceps biopsy would sample only normal mucosa, and EUS-TA was precluded as described above. As malignancy could not be excluded, resection was indicated for both diagnosis and treatment, and the operation was planned with both partial duodenectomy and subtotal stomach-preserving PD in view, with the final choice depending on the intraoperative findings.

**Fig. 1:**
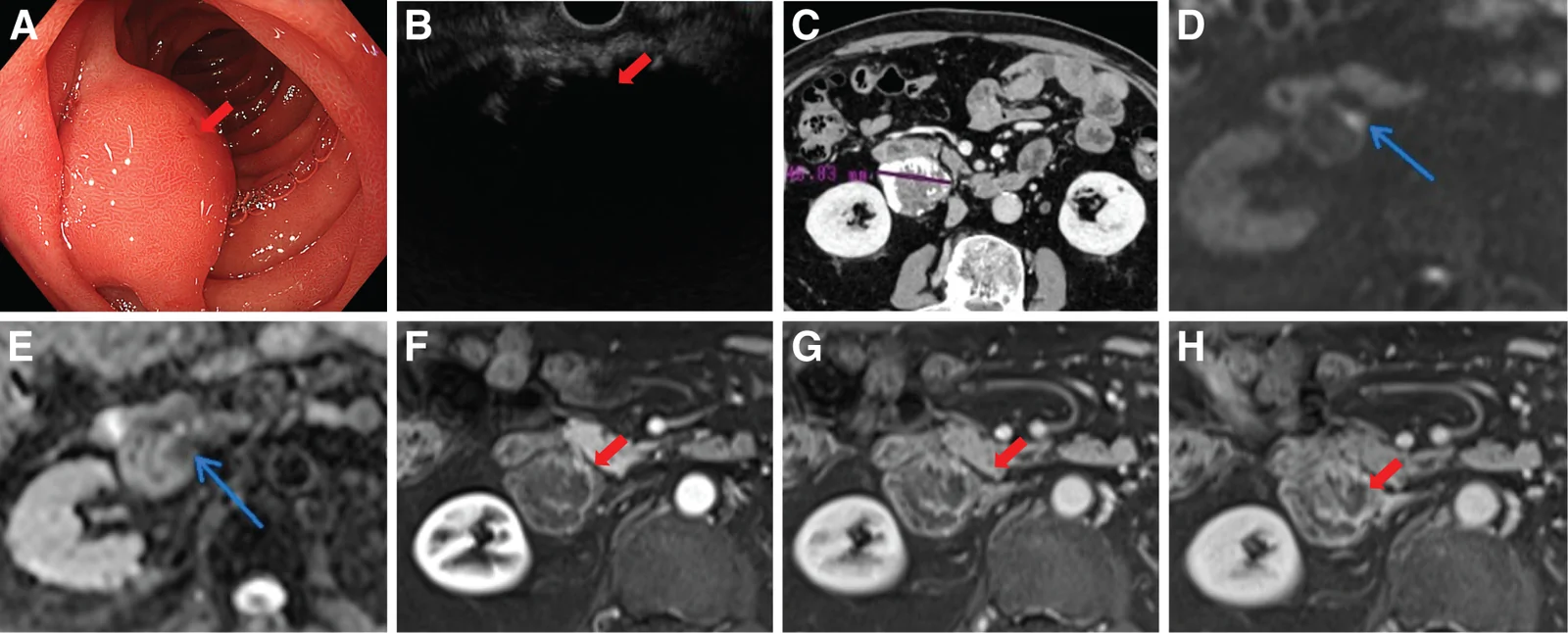
Imaging findings of duodenal amyloidosis. All images were obtained before surgery. On CT and MRI, the mass showed continuity with the pancreatic head arcade vessels, suggesting a mesenchymal tumor of duodenal origin, such as a GIST. (**A**) EGD. A submucosal tumor-like elevation with smooth overlying mucosa on the dorsal aspect of the descending duodenum. The intact overlying mucosa meant that forceps biopsy would have sampled only normal mucosa, and a preoperative tissue diagnosis could not be obtained. (**B**) EUS. Dense calcification produced acoustic shadowing (arrow), so the internal structure of the mass could not be evaluated and no safe puncture route could be identified. (**C**) Contrast-enhanced CT (venous phase, axial). A 58-mm exophytic mass with prominent peripheral calcification (arrow) on the dorsal aspect of the descending duodenum. (**D**) MRI, diffusion-weighted imaging. Faint hyperintensity at the periphery of the mass (arrow). (**E**) MRI, apparent diffusion coefficient map. Subtle hypointensity at the periphery of the mass (arrow). (**F**–**H**) Dynamic contrast-enhanced MRI obtained at 40 s (**F**), 70 s (**G**), and 180 s (**H**) after contrast injection. Progressive centripetal enhancement of the mass, spreading from the periphery toward the center (arrows). EGD, esophagogastroduodenoscopy; EUS, endoscopic ultrasonography; GIST, gastrointestinal stromal tumor

Preoperative laboratory tests showed normal hepatic function (AST 22 U/L, ALT 25 U/L), renal function (creatinine 0.88 mg/dL), and tumor marker levels (CEA 2.6 ng/mL, CA19-9 19.7 U/mL).

### Operative findings

Because a malignant mesenchymal tumor, such as a duodenal GIST invading the pancreatic head, could not be excluded, subtotal stomach-preserving PD was performed for diagnostic and therapeutic purposes. The operative time was 9 h 36 min, and the estimated blood loss was 1137 mL. Intraoperative findings demonstrated that the mass was located external to the muscularis propria of the duodenum, with an ill-defined border with the pancreatic head parenchyma (**Fig. 2A**). The tissue surrounding the mass showed chronic inflammatory change with marked neovascularization, and the retroperitoneal plane was obliterated. A laceration of the bile duct occurred intraoperatively. Because of concern regarding a postoperative biliary stricture, the anastomosis was taken down and refashioned. Postoperatively, a bile leak from the choledochojejunostomy developed but was managed conservatively with percutaneous drainage, and the patient was discharged on POD 32. The leak was minor and resolved with drainage alone, without the need for reoperation or any lasting clinical consequences.

**Fig. 2:**
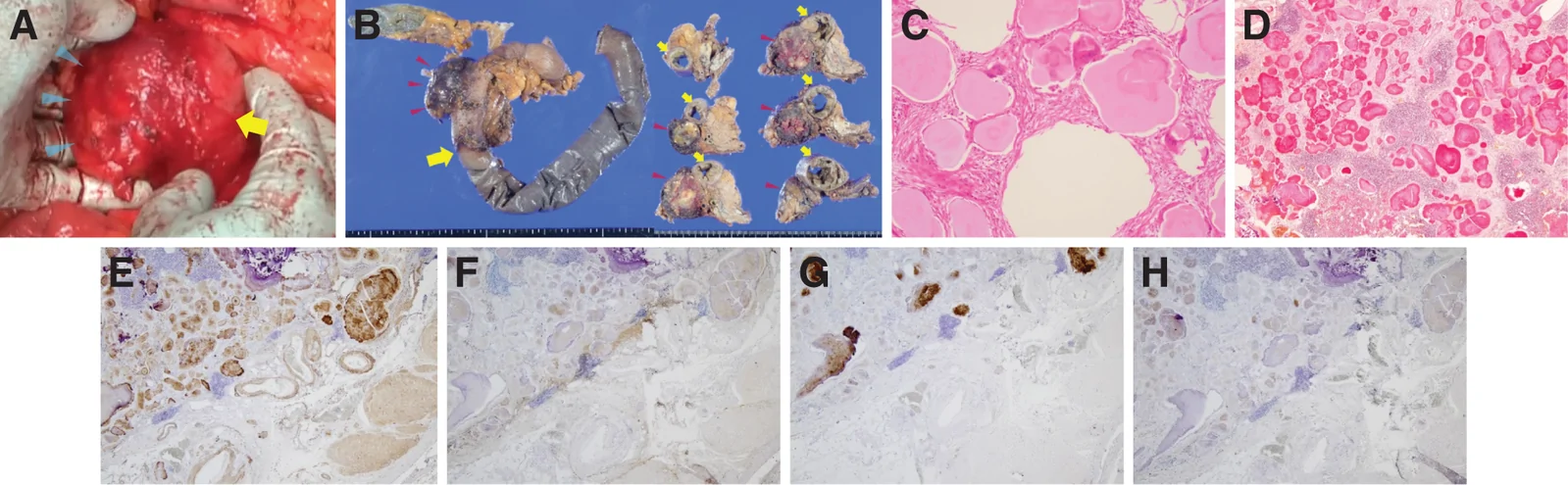
Gross and microscopic findings of duodenal amyloidosis. (**A**) Intraoperative photograph. The amyloid mass (blue arrowheads) on the dorsal aspect of the descending duodenum, with the duodenum indicated by the yellow arrow. The mass was located external to the muscularis propria and had an ill-defined border with the pancreatic head. (**B**) Gross appearance of the resected specimen. The left panel shows the gross photograph, and the right panel shows the cut sections. A firm, extensively sclerotic amyloid mass (red arrowheads) measuring 65 × 40 × 35 mm arises on the dorsal aspect of the descending duodenum and extends outward from the duodenal wall, with the duodenum indicated by the yellow arrows. (**C**) Congo red staining (original magnification ×100). Widespread deposition of eosinophilic amorphous material. Amyloid deposition was present in the lymph node interstitium, the duodenal muscularis propria, and the vascular walls. (**D**) DFS staining (×20). The deposits are positive (red). (**E**) Immunohistochemistry for λ light chain (×20). The deposits are positive. (**F**) Immunohistochemistry for κ light chain (×20). The deposits are negative. (**G**) Immunohistochemistry for ATTR (×20). The deposits are negative. (**H**) Immunohistochemistry for SAA (×20). The deposits are negative. The combination of λ positivity with κ, ATTR, and SAA negativity established the ALλ type. AL, amyloid light-chain; ATTR, amyloid transthyretin; DFS, direct fast scarlet; SAA, serum amyloid A

### Histopathological findings

Gross examination revealed a firm, extensively sclerotic mass measuring 65 × 40 × 35 mm on the dorsal side of the descending duodenum (**Fig. 2B**). The mass was continuous with the duodenal wall and projected exophytically into the retroperitoneum, external to the muscularis propria, whereas the overlying mucosa was smooth and intact, corresponding to the submucosal tumor-like elevation observed on EGD. Histologically, Congo red staining revealed widespread deposition of eosinophilic amorphous material (**Fig. 2C**). Marked amyloid deposition was also observed in the lymph node interstitium, duodenal muscularis propria, and vascular walls, with focal ossification and calcification. Because the deposition involved the muscularis propria and the surrounding perimuscular tissue, the lesion was considered to have arisen within the duodenal wall and extended outward, which is consistent with the intraoperative finding that the mass lay external to the muscularis propria. Histochemical staining with DFS was also positive (**Fig. 2D**). Immunohistochemical staining was positive for the λ light chain and negative for the κ light chain, ATTR, and SAA (**Fig. 2E**–**2H**). The diagnosis was localized ALλ-type amyloidosis. No neoplastic changes were observed in the pancreatic parenchyma or regional lymph nodes.

### Postoperative course and systemic workup

Comprehensive systemic evaluation after discharge included echocardiography, cardiac cine MRI, Holter electrocardiography, renal function tests, urinalysis, peripheral nerve examination, brain MRI, and random mucosal biopsies from upper and lower gastrointestinal endoscopies, all of which were within normal limits.

Postoperative investigations directed at excluding systemic amyloidosis and an underlying clonal or inflammatory disorder were unremarkable: serum free κ light chain, 48.8 mg/L; serum free λ light chain, 26.5 mg/L; κ/λ ratio, 1.84 (normal range); immunoglobulin G, 1112 mg/dL; IgA, 385 mg/dL; IgM, 55 mg/dL; and SAA, 2.3 mg/L. Autoantibody titers and complement levels were also within normal limits.

Serum and urine immunofixation electrophoresis showed no monoclonal protein. Systemic amyloidosis was excluded, and a final diagnosis of localized amyloidosis was established. No chemotherapy was administered, and the patient underwent regular follow-up. The patient continued follow-up on an outpatient basis.

## DISCUSSION

### Pathogenesis and classification of amyloidosis

The amyloid in the present case was of the ALλ type. AL amyloidosis results from the deposition of misfolded immunoglobulin light chains and may arise in association with a plasma cell dyscrasia or idiopathically, which is why demonstrating a monoclonal protein and excluding systemic disease were central to the management of this patient.^4)^

Gastrointestinal involvement is well described in systemic amyloidosis, whereas data on duodenal involvement in localized amyloidosis are limited to isolated case reports.^5)^ Localized AL amyloidosis is confined to a single organ and is occasionally encountered as a mass (amyloidoma); mass-forming lesions are usually of the AL type, although AA-type masses have also been reported.^6)^ In localized AL amyloidosis, the κ-to-λ ratio shows an organ-specific predilection, with λ predominating in the gastrointestinal tract, which is consistent with the ALλ type found in the present case.^7)^

### Diagnosis

The diagnostic difficulty in this case lay in obtaining tissue. EUS is ordinarily useful for identifying the layer of origin and guiding tissue acquisition, but extensive calcification can preclude evaluation of the internal structure and prevent identification of a safe puncture route, as it did here.^5)^ Imaging features such as the calcification pattern, enhancement dynamics, the absence of a fatty signal, and vascular continuity help narrow the differential diagnosis of a duodenal mass, but they are not pathognomonic for any single entity, and a mesenchymal tumor such as GIST cannot be distinguished from a mass-forming amyloidoma on imaging alone; therefore, tissue diagnosis remains essential. Once tissue was available, the diagnosis rested on Congo red staining, supported by DFS staining, and typing by immunohistochemistry. In AL amyloidosis, the detection of κ or λ light chains is essential.^8)^ Assessment of the κ/λ ratio in a localized lesion can also aid in the early detection of clonal plasma cell abnormalities.^7)^ Because a localized lesion cannot be distinguished from an early manifestation of systemic disease on histology alone, serum and urine immunofixation electrophoresis, free light chain assays, and evaluation of cardiac, renal, and neurological function were required to establish that the disease was localized.

### Treatment

Treatment depends on the type of amyloidosis and on symptoms. In localized AL amyloidosis, an asymptomatic, nonenlarging mass may be managed by observation, whereas surgical or endoscopic resection is considered for bleeding, obstruction, or when malignancy cannot be excluded^9)^—the last of which was the indication in the present case. Systemic AL amyloidosis, by contrast, is a plasma cell dyscrasia and requires chemotherapy^4)^; the distinction therefore has direct therapeutic consequences, and our patient was managed by observation alone after systemic disease had been excluded.

When PD is performed as a diagnostic and therapeutic procedure, it is critical to minimize complications such as lymphorrhea, blood loss, pancreatic fistula, and mortality. The extent of lymph node dissection should be minimized to the necessary extent. However, differential diagnoses for duodenal tumors include neuroendocrine tumors, duodenal carcinomas, lymphomas, and mesenchymal tumors such as GIST. For neuroendocrine tumors and duodenal carcinoma, current guidelines recommend lymph node dissection including stations #5 (suprapyloric), #6 (infrapyloric), #8a (anterior common hepatic artery), #12b/p (hepatoduodenal ligament), #13 (posterior pancreatic head), and #17 (anterior pancreatic head).^10–12)^ Extensive lymph node dissection is generally not required for lymphomas and GISTs. Therefore, imaging findings should be evaluated on a case-by-case basis to identify the most likely differential diagnosis and determine the optimal extent of lymph node dissection that balances oncological adequacy and complication risk. In general, the need for intraoperative frozen-section analysis of the pancreatic and bile duct margins is limited in the setting of diagnostic and therapeutic resection, although it should also be evaluated according to specific clinical scenarios. In the present case, the operation was prolonged, and blood loss was substantial because chronic inflammatory change with neovascularization around the mass had obliterated the retroperitoneal plane, and amyloid was demonstrated within the vascular walls of the lesion; the operative time was further prolonged because an intraoperative laceration of the bile duct, raising concern for postoperative biliary stricture, required the anastomosis to be taken down and refashioned. Comparable difficulty should be anticipated with mass-forming amyloid lesions at this site. The postoperative bile leak followed this reanastomosis and was therefore considered technical in origin rather than a consequence of amyloid deposition, which was confined to the lesion and regional lymph nodes, with systemic disease excluded; it was minor and resolved with drainage alone. Amyloid deposition may render tissue fragile and impair the local blood supply, and such changes have been implicated in impaired anastomotic healing in systemic amyloidosis. In the present case, however, deposition was confined to the lesion and the regional lymph nodes, no amyloid was identified in the bile duct used for the anastomosis, and systemic disease was excluded; a contribution from amyloid-related tissue fragility or impaired perfusion is therefore considered unlikely. This interpretation is necessarily cautious, as it rests on the pathological and operative findings of a single case.

### Previously reported cases of duodenal amyloidosis

To the best of our knowledge, only 4 cases of localized duodenal amyloidosis have been reported previously. (**Table 1**)^13–16)^ These included 1 case of an obstructive duodenal mass treated by endoscopic loop resection during endoscopic retrograde cholangiopancreatography, which proved to be AL-type duodenal amyloidosis^13)^; 1 case of ALκ-type amyloid deposition at the papilla causing common bile duct obstruction, treated with biliary stenting followed by systemic chemotherapy with cisplatin, vinblastine, and dacarbazine^14)^; 1 case of AA amyloidosis secondary to ulcerative colitis presenting with duodenal erosion, which improved with an increased corticosteroid dosage^15)^; and 1 case identified following gastrointestinal bleeding from duodenal erosions, subsequently diagnosed as ALκ-type amyloidosis associated with SMM, for which systemic therapy was initiated.^16)^ We searched PubMed using the terms “duodenal amyloidosis” AND “surgical resection” for articles published between 1980 and 2025 and identified no prior reports of PD performed for localized duodenal amyloidosis. Mass-forming lesions are extremely rare, and treatment strategies are determined on a case-by-case basis according to the patient’s symptoms and the presence of underlying diseases. Regarding amyloid type, 3 cases were AL type, and 1 was secondary AA type. Underlying conditions included SMM in 1 case and ulcerative colitis in 1 case, whereas the remaining cases were idiopathic. Regarding clinical presentation, cholangitis occurred in 1 case, and gastrointestinal bleeding occurred in 2 cases.

**Table 1:** Previously reported cases of duodenal amyloidosis

Reference	Age/Sex	Amyloid type	Underlying disease	Presentation	Treatment
Hauben et al., 1997^13)^	NR	AL	None	Duodenal mass causing obstructive symptoms	Endoscopic loop resection
Clough and Shah, 2015^14)^	61/F	ALκ	Primary amyloidosis	Common bile duct obstruction and cholangitis	Endoscopic biliary stenting and chemotherapy
Park et al., 2018 ^15)^	36/M	AA	Ulcerative colitis	Duodenal ulcers	Methylprednisolone for ulcerative colitis
Wagner et al., 2024^16)^	71/F	ALκ	SMM	Duodenal erosions and bleeding	Systemic therapy for SMM
Present case	71/M	ALλ	None	Asymptomatic; calcified mass on imaging	SSPPD

All listed cases are localized duodenal amyloidosis.

AA, amyloid A; AL, amyloid light-chain; ALκ, amyloid light-chain κ type; ALλ, amyloid light-chain λ type; F, female; M, male; NR, not reported; SMM, smoldering multiple myeloma; SSPPD, subtotal stomach-preserving pancreaticoduodenectomy

### Significance of long-term follow-up

The importance of long-term follow-up for localized AL amyloidosis can be considered from 3 perspectives. First, early detection of local recurrence after resection is essential; in localized AL amyloidosis of the head and neck, recurrence rates of 10%–12% within 4 years have been reported.^17)^ Second, monitoring for the development of clonal disease or plasma cell abnormalities is warranted. SMM is defined as the presence of serum M-protein ≥3 g/dL or 10%–60% clonal plasma cells in the bone marrow without end-organ damage, and it progresses to multiple myeloma at a rate of approximately 10% per year during the first 5 years.^18)^ Although no cases of direct progression from localized AL to SMM or systemic AL amyloidosis have been reported, periodic immunofixation electrophoresis and free light-chain assays can facilitate the early detection of de novo monoclonal proteins. Third, monitoring cardiac and renal function, peripheral nerve involvement, and the gastrointestinal tract is necessary in anticipation of a potential transition from localized to systemic disease. A recent comprehensive review reported that the risk of progression from localized to systemic AL amyloidosis is very low, with only 7 of 606 patients and 3 of 293 patients progressing in 2 large cohort studies^7)^; nevertheless, long-term surveillance is considered essential to safeguard the patient’s QOL. In the present case, in consultation with the hematology department, the follow-up plan included annual endoscopy, blood and urine tests, and echocardiography. Specifically, serum protein electrophoresis, serum-free light chains, quantitative immunoglobulins, hemoglobin, renal function, and serum calcium levels were monitored regularly, and a bone marrow examination was performed if any abnormality suggestive of a clonal plasma cell disorder was detected during follow-up. Further investigations, including initiation of chemotherapy, should be considered if recurrence, new monoclonal proteins, or organ dysfunction are detected. At the time of this report, the patient has remained recurrence-free for 2 years postoperatively.

### Clinical significance of the present case

The present case is, to our knowledge, the first report of PD for ALλ-type duodenal amyloidosis based on the aforementioned literature search. Based on previous reports, untreated duodenal masses may cause complications such as duodenal stenosis, hemorrhage, common bile duct obstruction, and pancreatic duct obstruction; therefore, even in asymptomatic patients, surgical resection should be considered when a localized mass cannot be distinguished from a malignancy. In patients deemed to be at high surgical risk because of advanced age or comorbidities, a staged approach combining less invasive diagnostic and therapeutic interventions, such as endoscopic hemostasis, biliary or pancreatic duct stenting, and surgical biopsy, followed by surgical intervention upon symptom onset or tumor enlargement, may represent a reasonable alternative strategy.

## CONCLUSIONS

We report a case of localized ALλ-type duodenal amyloidosis diagnosed after PD, which, to our knowledge, has not been previously reported. When preoperative tissue diagnosis of a calcified duodenal mass is not feasible, and malignancy cannot be excluded, surgical resection may serve as both a definitive diagnostic and therapeutic approach. After diagnosis, exclusion of systemic amyloidosis and long-term surveillance for recurrence and clonal progression are essential.

This case highlights the importance of considering localized amyloidosis in the differential diagnosis of calcified duodenal masses.

## DECLARATIONS

### Availability of data and materials

Data for this study are not publicly available because of concerns regarding patient confidentiality but are available from the corresponding author upon reasonable request.
